# Supplementary material for: Protective Human Anti-Poxvirus Monoclonal Antibodies Are Generated from Rare Memory B Cells Isolated by Multicolor Antigen Tetramers
Source: Vaccines (Basel). 2022 Jul 6;10(7):1084. doi: 10.3390/vaccines10071084 (PMC9319751; doi:10.3390/vaccines10071084)
Supplement: Supplementary file 1 [file vaccines-10-01084-s001.zip › vaccines-1785043-supplementary.pdf]

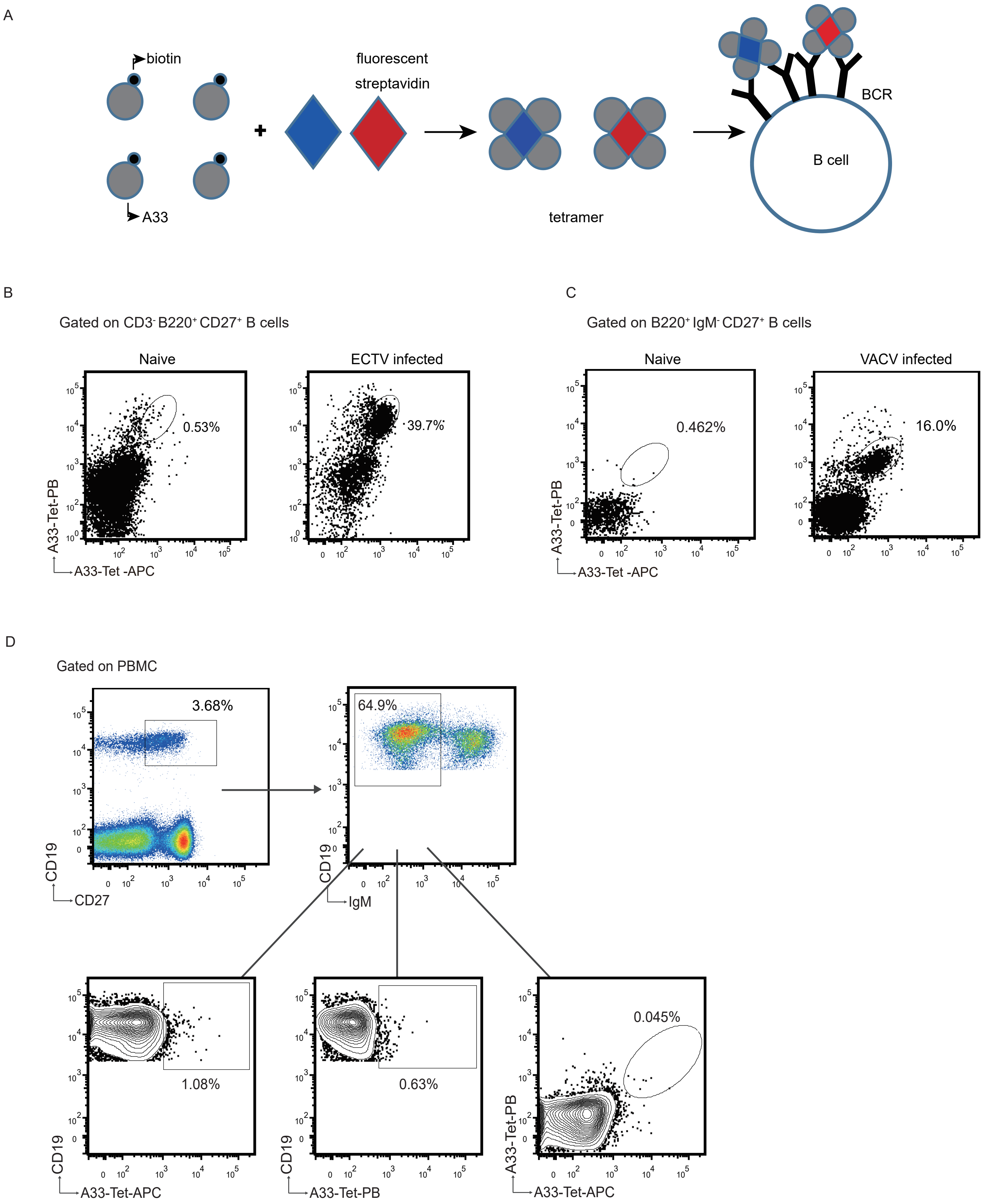

Figure.S1 Generation of A33 tetramer and tetramer-based screening of antigen-specific B cells. (A) Scheme of A33 tetramerizaion and B cell staining. Biotinylated A33 was incubated with different fluorescently labeled streptavidin to generate tetramers, respectively. Multiple A33 tetramers were used together along a panel of mAbs to indentify specific memory B cells. (B-C) Detection of A33-specific B cells in mice. (B) B6 mice were infected with 3000 PFU ECTV in the footpad. Splenocytes were isolated from ECTV infected mice at 4-5 weeks pi and stained for A33 specific B cells. Splenocytes from naive mice were used as control. The cells were first gated on $CD3^{-}$ $B220^{+}$ $CD27^{+}$ B cells, then A33-Tet-PB and A33-Tet-APC double positive B cells were identified. Numbers adjacent to gate represent the percentage of A33 tetramers positive cells within the gated B cells. (C) B6 mice were i.p. infected with 5×10^{6} PFU VACV. Splenocytes were isolated from VACV infected mice at 4-5 weeks pi and stained for A33 specific B cells. Splenocytes from naive mice were used as control. The cells were first gated on $B220^{+}$ IgM^{-} $CD27^{+}$ class switched memory B cells, then A33-Tet-PB and A33-Tet-APC double positive B cells were identified. Numbers adjacent to gate represent the percentage of A33 tetramers positive cells within the gated B cells. (D) Sorting of single A33-specific memory B cells from PBMC of a healthy volunteer. Identification of single A33 tetramer positive B cells or multicolor A33 tetramers positve B cells were shown in the gated $CD19^{+}$ $CD27^{+}$ IgM^{-} class switched memory B cells. Frequency of each cell population is shown beside the population.

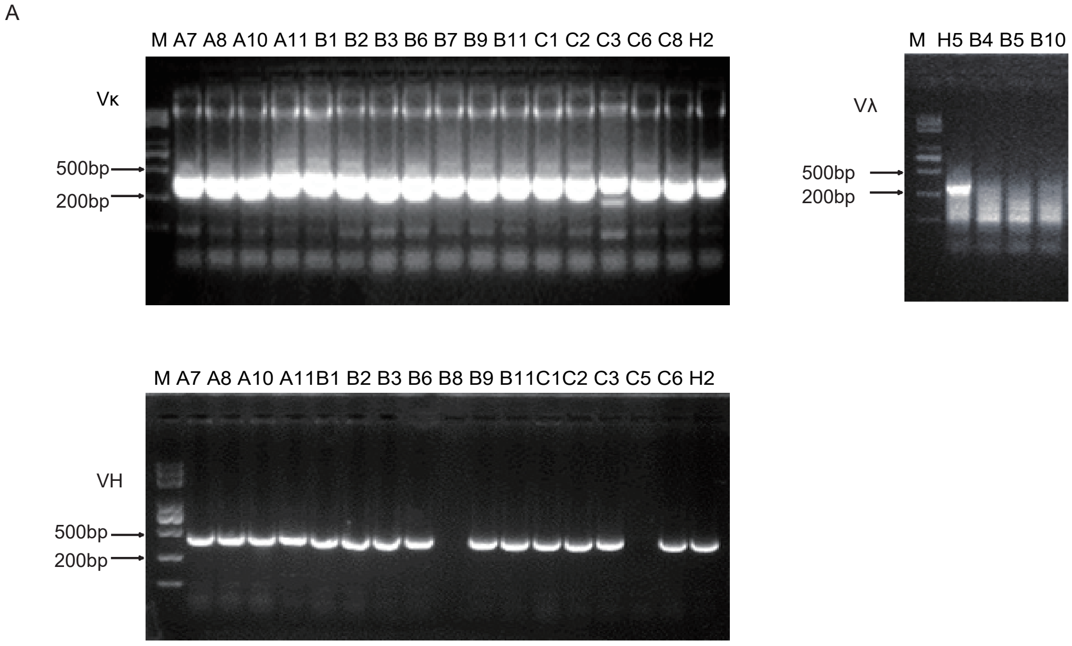

B

	V-Gene and allele		J-Gene and allele	
IgH	14	IGHV3-7*05	14	IGHJ5*01
	4	IGHV3-23*04	3	IGHJ4*02
	1	IGHV3-33*01	1	IGHJ5*02
			1	IGHJ3*02
IgK	11	IGKV1-5*03	11	IGKJ2*01
	8	IGKV1-12*01	6	IGKJ1*01
			2	IGKJ5*01

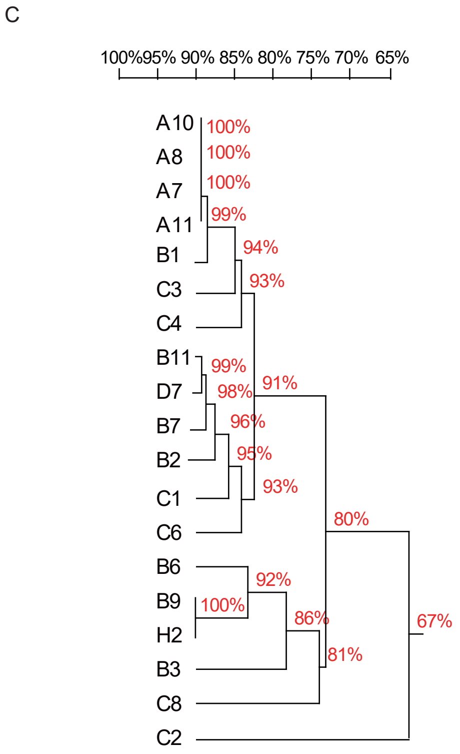

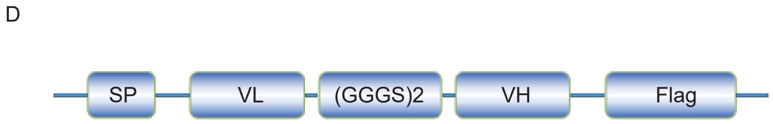

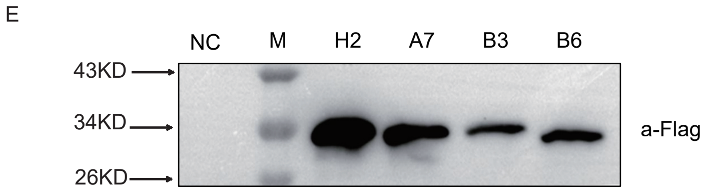

Figure.S2 Amplification of Ab variable region and construction of ScFvs. (A) VH and VL genes were amplified by nested RT-PCR from sorted single cell cDN Analyzed on a 2% agarose gel and cloned into pcDNA3.1 plasmid. (B) V, J regions of the 19 Abs were analyzed by IMGT. (C) DNAMAN software compared the sequence homology of paired VH and VL. (D) Scheme of consturct for scFv. (E) 293F cells were transfected with vector (negative control) or the indicated scFv plasmids. After 48 hours, the expression of scFvs was determined in the cell supernatant by western blot using the anti-flag antibody.

Extended Materials and Methods

Cells, viruses

293T cells (ATCC, CRL-3216) and BSC-1 cells (ATCC, 3168) were cultured in DMEM medium supplemented with 10% FBS, 2 mM L-glutamine, 100 μm nonessential amino acids, 10 mM HEPES buffer, 100 IU/ml penicillin, 100 μg/ml streptomycin (all from Gibco), and 0.05 mM β-mercaptoethanol (Amresco). 293-F cells (Invitrogen, R790-07) were cultured in SMM 293-TI (Sino-Biological). All cells were cultured at 37°C in 5% CO_2.

Initial stocks of ECTV Moscow and VACV Western Reserve were obtained from Dr. Luis Sigal (Thomas Jefferson University) and amplified and quantified as described (23). For the determination of virus titers in mice spleens, the spleens were made into a single cell suspension between two frosted slides and resuspended in 5 ml complete RPMI medium. 500μl of the cell suspensions were frozen and thawed three times and titers were determined in 10-fold serial dilutions of the cell lysates. Virus titers were calculated as PFU/spleen. To determine the virus titers in liver, a portion of the liver was weighed and homogenized in the medium using a tissue homogenizer and titrated.

Production and Biotinylation of A33

The coding sequences for the extraviral domain of VACV Western Reserve A33 (24) were amplified by PCR from genomic DNA. For A33 the forward and reverse primers used were 5'AAACCATGGGCCATCACCATQCACCATCACTGCATGTCT-GCTAACGAGGCTG3' and 5'AAAGGATCCGTTCATTGTTTTAACACAAAAATA CTTTC3', respectively. To facilitate purification, the 6×His tag was fused to the N-terminus. Avi-tag (GLNDIFEAQKIEWHE)(30) was fused to the C-terminus of A33 to mediate biotinylation. The whole construct was cloned into the PET-28a(+) vector and transformed into DH5α competent cells. The expression vector was verified by DNA sequencing and then transformed into BL21 (DE3) competent cells for expression. The transformed BL21 (DE3) cells were grown overnight at 37°C and

inoculated at 10% into LB medium. The culture was grown at 37°C until the OD reached 0.6-0.8, IPTG (final concentration 0.4 mM) was added to induce protein expression and cells were harvested 4h later and lysed by sonication. The inclusion bodies were pelleted by centrifugation for 20 min at 8000×g, washed with washing buffer (20 mM Tris–HCl pH 8.0, containing 1% Triton-X100) followed by distilled water to remove contaminating salts and detergents.

Recombinant A33 was purified under denaturing conditions by Ni-NTA metal affinity chromatography. The inclusion bodies were solubilized with 8M urea and loaded onto Ni-NTA agarose (Thermo, QB211702), according to the manufacturer's recommendations. After washing out the unbound proteins, the target protein was eluted by 0.5M imidazole in 8M Urea lysis buffer. The purified proteins were refolded by dialysis against PBS (phosphate-buffered saline). Protein concentrations were determined using a bicinchoninic acid assay (EpiZyme, ZJ102) with bovine serum albumin as a standard. The purity of each protein was confirmed by sodium dodecyl sulfate-polyacrylamide gel electrophoresis (SDS-PAGE).

Biotinylation of purified A33 protein was performed by using the biotin-protein ligase kit (GeneCopoeia™, B1001) according to the manufacturer's instruction. Biotinylation of the protein was verified by Western Blot using the HRP conjugated streptavidin (Earthox, E030100).

The coding sequences for the extraviral domain of A33 from VARV or MPXV with N terminus 6×His tag and C-terminus Avi-tag were synthesized by GenScript and directly cloned into the PET-28a(+). The purification and biotinylation of VARV/MPXV A33 were the same as that of VACV A33.

Constructing and expression of single chain antibody (scFv)

The scFvs were constructed by connecting paired V_L and V_H with a $(GGGS)_2$ linker, an Ig leader sequence (METDTLLLWVLLLWVPGSTGD) was used as a signal peptide at the N-terminus of scFv to mediate the secretion of the scFv into the cell supernatant. A flag tag was fused to the C-terminus of scFv for easy detection. The constructs were cloned into pcDNA3.1 vector (Invitrogen, V79020). The constructed

scFvs were verified by sequencing. The plasmids were transfected into 293F cells with Polyethylenimine (PEI), and the cell supernatants were collected after 48-72 hours.

Production of H2 IgG

H2 IgG was generated by combining V_H and V_L with constant region of IgG1 heavy and light chain, respectively. The plasmids were synthesized by GenScript. The constructed heavy and light chain plasmids were co-transfected into 293F cells. The supernatant was collected and H2 IgG was purified by protein A (Nanomicro, UniMab 50) according to the manufacturer's recommendations. The purified protein was further dialyzed with PBS. Protein concentration was determined using a bicinchoninic acid assay (EpiZyme, ZJ102) with bovine serum albumin as a standard. The protein was stored in aliquots at -80°C.

ELISA

96-well bottom ELISA plates were coated with VACV A33, VARV A33 (50 μg/ml), ECTV (1×10^7 PFU/ml), VACV (1×10^7 PFU/ml), PR8 (1×10^7 PFU/ml) or BSA (50 μg/ml) at 4°C overnight as indicated. Plates were blocked for 2 h at 37°C with PBS containing 5% BSA. The plate was then incubated with the indicated antisera, cell culture supernatant or H2 IgG at 37°C for 2 hours. The plates were washed three times with PBS containing 0.05% Tween-20. For detection of sera, HRP-conjugated goat anti-mouse IgG (Jackson ImmunoResearch, 115-035-003) were added to each well and incubated for 1 h at 37°C. For detection of scFv, anti-flag M2 mAb (Sigma-Aldrich, F1804) were added to each well and incubated at 37°C for 2 h, and then washed five times. Then HRP-conjugated goat anti-mouse IgG (Jackson ImmunoResearch, 115-035-003) antibodies were added to each well and incubated for 1 h at 37°C. For detection of H2 IgG, HRP goat anti-human IgG (EARTH, E030170-02) were added to each well and incubated for 1 h at 37°C. After incubation with the HRP-conjugated antibodies, the plates were washed five times. TMB 1-Component Microwell Peroxidase Substrate (KPL) was added to each well and the

plates were incubated at room temperature for 5-20 min. The reactions were stopped by adding 50μl 0.5 M H_2SO_4. The optical density (OD) at 450 nm was determined by Thermo MULTISKAN FC.

Comet inhibition assay

Confluent BSC-1 cells in 6 well plates were infected with ECTV (MOI=0.01) in 0.5 ml RPMI medium with 2.5% FBS (RPMI 2.5). The media containing virus was aspirated after 1h incubation at 37°C, and the cells were overlaid with 1.5 ml RPMI 2.5 containing the indicated dilutions of antisera or antibodies. Cells were incubated for 5 days at 37°C and stained with crystal violet as described for virus titers.

Surface plasmon resonance analysis

The affinity between H2 and A33 was measured at room temperature using a Biacore T100 system with CM5 chips (GE Healthcare). The immobilization of H2 was carried out following the canonical amino coupling chemistry, operating at a flow rate of 30 μL/min and injection for 60 seconds, dissociation for 180 seconds. A33 in varied concentrations (from 1μM to 62.5 nM, repeat at 250 nM) were allowed to flowover sensor surface in a running buffer of phosphate-buffered saline (PBS, pH 7.4) containing 0.01% (v/v) Tween-20. The binding affinity KD (equilibrium dissociation constant, KD = Kd/Ka, where Kd and Ka represent the dissociation rate constant and association rate constant, respectively) values were obtained using a series of A33 concentrations. The affinity was calculated using a 1:1 (Langmuir) binding fit model with BIA evaluation software.

The V_H and V_L Sequences of mAbs

A7 mAb:
V_K:
DILMTQSPSTLSASVGDRVSITCRASQFISGWLAWYQQKPGKAPRLLIYRASSL
QSGVPSRFSGSGSGTEFTLAINSLQPDDFATYYCQQYDTYPYTFGQGTIL

V_H:
EVQLVESGGGLVQPGGSLRLSCAASGFNLINIWMSWVRQTPGKGLEWVANIN
HDESGKYVVDSVKGRFSVSRDNSKHSLYLQMNTLRPEDTAVYYCARSSGGYF
DSWGQGIQVTVSS

H2 mAb:
V_K:
DIQLTQSPSSLSASVGDRVTITCRPSQGVSRWLAWYQQKPGKAPKFLIYAASSL
QSGVPSRFSGSGSGTDFTLTINSLQPEDFATYYCQQANSFPWTFGQGTKVEIK

V_H:
EVQLVQSGGGLVQPGGSLRLSCAASGITFSTYAMSWVRQAPGKGLEWVSAIG
GSGSRTYYGDSVKGRFTISRDNSKNTLYLQMNSLRAEDTAIYYCAKVFRDSSG
YYGGFDDWGQGTLLTVSS
